# Long-term monitoring of Graves’ disease in children and adolescents: a single-center experience

**DOI:** 10.3906/sag-1804-177

**Published:** 2019-04-18

**Authors:** Selma TUNÇ, Özge KÖPRÜLÜ, Hatice ORTAÇ, Özlem NALBANTOĞLU, Ceyhun DİZDARER, Korcan DEMİR, Behzat ÖZKAN

**Affiliations:** 1 Clinic of Pediatric Endocrinology, Dr. Behçet Uz Children’s Hospital, İzmir Turkey; 2 Department of Biostatistics, Faculty of Medicine, Dicle University, Diyarbakır Turkey; 3 Department of Pediatric Endocrinology, Faculty of Medicine, Dokuz Eylül University, İzmir Turkey

**Keywords:** Graves’ disease, hyperthyroidism, antithyroid drugs

## Abstract

**Background/aim:**

Graves’ disease (GD) is more severe, requires a more complex treatment, and has a lower probability of achieving remission in children than in adults. There is no consensus on the appropriate duration of antithyroid drug (ATD) treatment. Surgical or radioactive iodine (RAI) treatments are not definitive and generally result in permanent hypothyroidism. This study’s goal was examining the effectiveness of ATD treatment in children and adolescents with GD and determining the risk factors of remission and relapse.

**Materials and methods:**

This retrospective study included 45 patients (36 females and 9 males, median age 12.5 years) aged 4–18 who were diagnosed with GD between 2003 and 2017. All patients initially were treated with an ATD. ATD treatment was discontinued at a mean of 23.2 ± 13.2 months (10–37 months).

**Results:**

Patients were classified into remission (n = 24) and relapse groups (n = 21). The duration of initial ATD treatment in the remission group was longer (26.91 ± 5.17 months) than in the relapse group (19.09 ± 7.14 months) (P = 0.01). The total ATD treatment duration was statistically longer in the remission group (42.14 ± 14.35 months) than in the relapse group (26.95 ± 16.13 months) (P = 0.03).

**Conclusion:**

Long-term initial ATD treatment and long-term total ATD treatment were evaluated as positive parameters for the remission of Graves’ disease in children and adolescents. Our findings showed that the chance of long-term remission increases in direct proportion to the initial ATD treatment duration and the total ATD treatment duration.

## 1. Introduction 

Graves’ disease (GD) is the most common cause of hyperthyroidism and constitutes 10%–15% of thyroid diseases in children. The incidence of GD is about 1/10,000 in children and adolescents (1–3). GD is more common in girls (F/M = 5/1) and peaks in adolescence at the age of 11–15 years (4). The diagnosis is made with increased serum-free T3 (fT3) (2.6–4.8 pg/mL) and free T4 (fT4) (0.7–2 ng/dL) levels, suppressed serum TSH level (0.4–3.0 µIU/mL), positive thyroid receptor antibody (TRab) (<1.5 IU/L), and/or the presence of exophthalmos (4,5).

There is no consensus regarding the optimal treatment method for GD in children and adolescents. Treatment priorities may differ according to age (4,6). Since there is no wide-scale, prospective, randomized study results related to the treatment of GD in children and adolescents, treatment of GD is generally planned individually (1). 

There are medical, surgical, and radioactive iodine (RAI) options for the treatment of GD. Each treatment option has its own risks. In general, antithyroid drugs (ATDs) are the first preference in the treatment of GD (propylthiouracil (PTU), methimazole (MMI)) (7). Two ATD regimens for medical treatment of GD have been used: dose titration and blockage replacement. In dose titration, the usual starting dose is 15–30 mg/day of methimazole (or equivalent doses of other thionamides). The daily dose is tapered down to the lowest effective dose, avoiding both hyperthyroidism and hypothyroidism. Thyroid function is checked every 4–6 weeks for the first 4–6 months, then every 3–4 months until treatment is stopped after 18–24 months. In blockage-replacement, persistently high ATD doses are given in association with L-thyroxine replacement to avoid hypothyroidism. Higher ATD doses may provide a greater immunosuppressive effect for permanent remission. Avoidance of hypothyroidism and hyperthyroidism seems easier than with the dose-titration method, treatment duration is shorter, and fewer visits are required. However, the much higher number of tablets taken every day may create compliance problems and may have a higher risk of side effects (8).

Long-term use of ATDs may lead to the development of side effects and adaptation problems (7,9). The mortality rate is approximately 25%–50% after the development of PTU-related hepatoxicity. Another study showed that severe liver damage occurred in 22 patients treated with PTU, resulting in high rates of mortality (death in 9 cases, liver transplantation in 5 cases). In 2009, the US Food and Drug Administration (FDA) recommended that PTU should not be used to treat GD in children and adolescents (10–13). MMI treatment is available as an alternative treatment since it has comparatively fewer side effects. Limitations in medical treatment (toxicity, relapse, adaptation problems, treatment duration) place RAI or surgical treatment at the forefront (4,6,7). RAI and surgical treatments, however, bear risks in terms of timing of the procedure, age of the patient, and some potential complications such as hypothyroidism.

Studies that investigate the predictive factors for relapse or remission and the requirement for radical treatments at an early stage are limited. The course of GD varies individually, as do the factors associated with remission and relapse. Patients who are older, have a higher body mass index, smaller goiters, lower thyroid hormone levels at diagnosis, and negative TRab can achieve remission in the early period. It has been shown that prepubertal children can achieve remission for a longer period than pubertal and postpubertal children. The risk of relapse increases in patients whose TRab levels are higher. Patients whose hyperthyroidism was corrected within 4–6 months with ATD treatment achieved long-term remission. Management of patients with GD will be easier if patients can be identified as requiring long-term ATD treatment or requiring radical treatment (RAI or surgery) through reliable indicators for relapse and remission in the early period (14,15). 

The objective of this study was to evaluate the clinical features and to determine the predictive factors related to relapse and remission in children and adolescents with GD.

## 2. Materials and methods 

This retrospective study included 45 subjects with GD who were aged 4–18. The patients were followed for at least 1 year between 2003 and 2017 at the Pediatric Endocrinology Department of Behçet Uz Children’s Hospital. The study was approved by the Behçet Uz Children’s Hospital’s Ethical Council with ethical council number: 2016 / 03-01, folder number: 2016/61. 

The following parameters were recorded for each patient at the time of admission: age at diagnosis, sex, body weight, weight standard deviation score (SDS), height SDS, body mass index SDS, pulse rate, systolic and diastolic blood pressure (above the 95th percentile was accepted as hypertension for the norms determined for age and sex (16)), clinical goiter and ophthalmopathy, puberty state according to Tanner staging (17,18), and family history of autoimmune thyroid disease. Free T3, free T4, fT3/fT4 ratio, TRab, antithyroid peroxidase antibody (anti-TPO) (0–9 IU/mL), and antithyroglobulin antibody (anti-TG) (0–4 IU/mL) were recorded at the onset of diagnosis. High transaminase level was considered when alanine aminotransferase (ALT) (0–35 U/L) and aspartate aminotransferase (AST) (0–35 U/L) were twice the normal values, and leukopenia was considered when the leukocyte count was below 4500/µL (16). Thyroid volume, thyroid parenchyma echogenicity, pseudonodular appearance, and blood flow rate evaluated via thyroid ultrasonography were recorded. Thyroid gland volume and volume SDS were calculated using ÇEDD Çözüm software (TPEDS Metrics) (19,20). 

GD was diagnosed with increased fT3 and fT4 levels, suppressed TSH level, and/or positive TRab (2,21,22). The patients were monitored both clinically and with laboratory testing for 3–6 months. Starting dose, duration of use, side effects of the antithyroid drugs (MMI, PTU, propranolol), and periods of normalization of fT4 and TRab levels were recorded. The patients who had received RAI or surgical treatment (total or subtotal thyroidectomy) were registered. The final states of the cases were classified as hypothyroidism, euthyroidism, and currently undergoing ATD treatment. 

Remission was defined as the sustainment of a clinical and biochemical state of euthyroidism for at least 1 year following the termination of ATD treatment and no relapse in follow-up (23,24). Relapse was defined as increased fT4 or fT3 levels in addition to suppressed serum TSH level following dose reduction or termination of ATDs in patients who receive at least 12 months of proper treatment (2). The cases were classified into the groups “relapse” and “remission.”

### 2.1. Statistical analysis 

SPSS 24.0 for Windows was used for statistical analysis. Descriptive statistics, number and percentage for categorical variables, and average ± standard deviation for data that meet the normal distribution parameters for numeric variables have been provided. Student’s t-test was applied for continuous variables (age at diagnosis; anthropometric measurements; mean initial fT4, fT3, and fT3/fT4 ratio; initial thyroid US volume; fT4 normalization time; TRab normalization time; initial ATD treatment duration; and total duration of ATD Rx) during the comparison of 2 independent groups, while the chi-square test was used for categorical variables (sex; family history of AITD (n); puberty (n); Graves’ ophthalmopathy (n); initial positive TRab (n); initial positive anti-TPO (n); initial positive anti-TG (n); the number of patients who initially received MMI, PTU, and Na-L-thyroxine; and positive TRab at the end of ATD Rx (n)). P < 0.05 was accepted as significant. 

## 3. Results 

Of the 45 patients included in the study, 36 (80%) were female and 9 (20%) were male. Their median age at diagnosis was 12.5 years (range: 4–18 years). Thirty-two (71%) were in the pubertal stage. Family history of autoimmune thyroid disease was present in 32 (72%) cases. Ophthalmopathy was determined in 11 (24%) of the cases. Systolic blood pressure, defined as above the 95th percentile according to sex and height, was detected in 4 (13%) out of 30 patients whose blood pressure (BP) was measured. Body weight SDS and BMI SDS of these patients were within normal limits. The mean weight SDS was –0.10 ± 0.9 and the mean BMI SDS was 0.40 ± 0.89.

**Figure 1:**
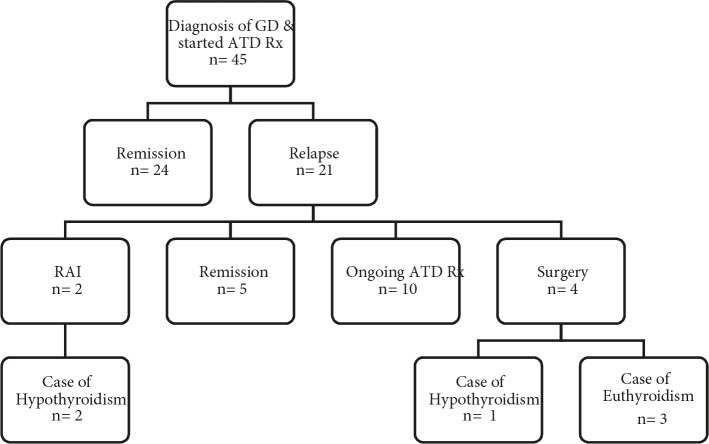
Clinical course of cases.

**Figure 2:**
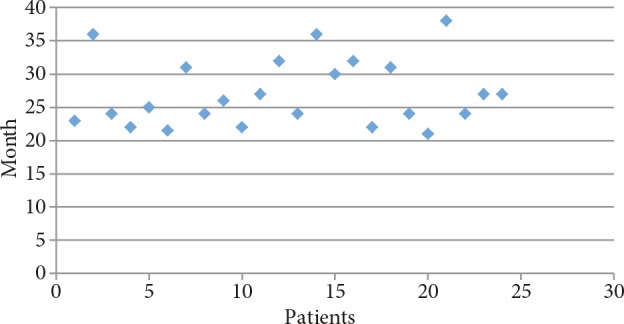
Initial ATD treatment duration in the remission group.

**Figure 3:**
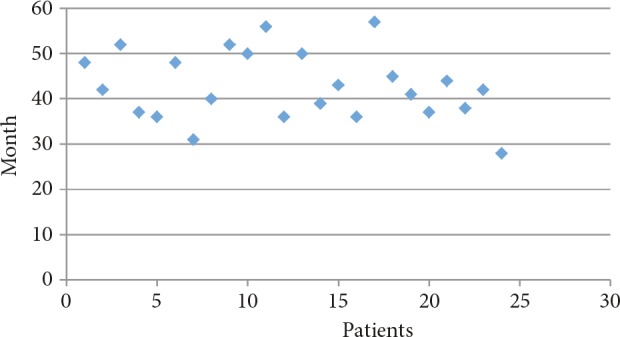
Total ATD treatment duration in remission group.

Thyroid gland size was observed in physical examination to be increased above average for age and sex in 38 patients (85%). The mean thyroid volume and the mean thyroid volume SDS calculated via thyroid ultrasonography were 14.8 ± 8.6 mL and 7.86 ± 6.61 mL. Mean fT4 level was 3.96 ± 1.67 ng/dL, mean fT3 level was 14.46 ± 5.94 pg/mL, and mean fT3/fT4 ratio was 3.65 ± 0.72 at the time of diagnosis. At the onset of diagnosis, TRab was measured in 38 patients. TRab was detected positive in 26 (68%) patients and was detected negative in the remaining 12 patients (9 relapse, 3 remission). In these 12 patients and in 7 patients whose TRab was not measured at the time of diagnosis, TRab was detected positive in the follow-up period. For this reason, hashitoxicosis was not considered. Anti-TPO and anti-TG levels were positive in 24 (63%) cases. TRab, anti-TPO, and anti-TG were positive in 14 (31%) of cases. TRab was detected positive only in 6 (13%) cases. Of the 12 cases with negative TRab levels, positive anti-TPO and/or anti-TG levels were detected in 7 (16%). Initially, ATD treatment was started for all patients diagnosed with GD. Twenty-two of the patients were treated with MMI (mean starting dose: 0.65 ± 0.24 mg/kg/day), and 23 of the patients were treated with PTU (mean starting dose: 3.88 ± 1.37 mg/kg/day). Following the FDA recommendation in 2009, PTU treatment was discontinued in 5 patients and MMI therapy was started instead. Initially propranolol (mean starting dose: 1.50 ± 0.46 mg/kg/day) was started in 36 patients (80%) because of tachycardia. Dose-titration treatment was used in 9 (20%) of the cases, and blockage-replacement treatment was used in 36 (80%) of the cases. ATD treatment was stopped for patients with normal or close to normal TRab levels as well as those who remained in euthyroid state for at least 12 months with low doses of ATD (MMI 2.5–5 mg/day or PTU 25–50 mg/day). ATD treatment was discontinued at the end of a mean treatment period of 23.2 ± 13.2 months (10–73 months) in all cases. Figure 1 shows the clinical course of the cases. Remission was achieved by 24 (53%) patients during follow-up. Relapse occurred in 21 (47%) cases. Of the 21 cases of relapse, 2 of these patients received RAI treatment and 4 underwent surgical treatment (three subtotal and one total thyroidectomy). ATD treatment was restarted in these remaining 15 patients. Of these 15 patients, 10 (22%) are still undergoing ATD treatment and 5 went into remission during follow-up. In the long term, 32 patients achieved euthyroidism (71%), hypothyroidism developed in 3 cases (1 case after total thyroidectomy, 2 cases after RAI), and 10 (22%) patients were still under ATD treatment (Figure 1). There was no statistically significant difference between remission and relapse groups according to age at diagnosis, weight SDS, height SDS, and BMI SDS (Table). Thyroid function tests and thyroid volume of the relapse group were determined to be similar in comparison with the remission group (P = 0.142). The time until TRab reached a normal level was not statistically significant even though there was an increase in the relapse group (28.8 ± 11 months) compared to the remission group (22.3 ± 13.02 months) (P = 0.267). The period of total ATD treatment was higher in the remission group (42.14 ± 14.35 months) in comparison with the relapse group (26.9 ± 16.1 months) (P = 0.03). The duration of initial ATD treatment was statistically higher in the remission group than in the relapse group (respectively 26.9 ± 5.17 months and 19.09 ± 7.14 months, P = 0.01). Figure 2 shows the distribution of the initial ATD treatment duration in the remission group. Figure 3 shows the distribution of the total ATD treatment duration in the remission group. Dose-titration treatment was administered in 3 cases in the remission group and 6 cases in the relapse group. Blockage-replacement treatment was used in 21 cases in the remission group and 15 cases in the relapse group. There was no significant difference between dose-titration method and blockage-replacement method with regard to remission and relapse development (P = 0.179).

**Table:** Clinical and biochemical characteristics of remission and relapse groups. Student’s t-test and chi-square test were used for statistical analysis.

		Remission (n = 24)	Relapse (n = 21)	P
Age at diagnosis (years)		10.96 ± 3.24	12.01 ± 3.22	0.246
Sex (F/M)		18/6	18/3	0.370
Weight SDS at diagnosis		0.01 ± 0.87	–0.24 ± 1.03	0.375
Height SDS at diagnosis		0.39 ± 1	0.42 ± 0.77	0.911
BMI SDS at diagnosis		–0.38 ± 1.08	–0.31 ± 1.22	0.839
Family history of AITD (n)		15	17	0.173
Puberty (n)		15	17	0.173
Graves’ ophthalmopathy (n)		7	4	0.431
Initial fT4 (0.7–2 ng/dL)		3.66 ± 1.69	4.20 ± 1.73	0.302
Initial fT3 (2.6–4.8 pg/mL)		13.32 ± 6.64	14.86 ± 5.61	0.410
Initial fT3/fT4 ratio		3.66 ± 0.76	3.63 ± 0.70	0.892
Initial positive TRab (n)		10	16	0.064
Initial positive anti-TPO (n)		8	16	0.060
Initial positive anti-TG (n)		12	12	0.580
Initial thyroid US volume (mL)		16.46 ± 6.62	12.82 ± 9.98	0.142
Initial	PTU (n)	16	7	0.080
	MMI (n)	8	14	0.186
Na- L-thyroxine (n)		21	15	0.179
Positive TRab at end of ATD Rx (n)		2	3	0.602
fT4 normalization time (weeks)		6.45 ± 3.89	6.85 ± 4.36	0.731
TRab normalization time (months)		22.28 ± 13.02	28.85 ± 11.00	0.267
Initial ATD treatment duration (months)		26.91 ± 5,17	19.09 ± 7.14	0.01
Total ATD treatment duration (months)		42.14 ± 14.35	26.95 ± 16.13	0.03

AITD: Autoimmune thyroid disease, BMI: body mass index, SDS: standard deviation scores, TRab: thyrotropin receptor antibody, TPO: thyroid peroxidase, TG: thyroglobulin, US: ultrasonography, ATD: antithyroid drug, fT4: free thyroxine, fT3: free triiodothyronine, PTU: propylthiouracil, MMI: methimazole, RAI: radioactive iodine.

Side effects due to ATD treatment were observed in 2 cases (4.4%). One patient had elevated transaminase levels, while the other had an allergic skin rash. Both were receiving PTU at that time. PTU treatment was changed to MMI because of side effects. The mean age at diagnosis of patients who developed side effects was 12.5 ± 3.5 years.

## 4. Discussion 

Long-term remission is observed at lower rates for GD in children in compared to adults. While the remission rate in children and adolescents in response to ATD treatment varies from 29% to 49%, the rate is between 39.5% and 85% in adults. Ohye et al. (25) reported that the remission rate was 46.2% and the relapse rate was 34.2% in children and adolescents. Remission and relapse rates were determined to be 53.4% and 46% in our study.

The onset of GD is thought to depend on complex interactions among multiple genetic, environmental, and endogenous factors. For this reason, the factors associated with remission and relapse may also vary from individual to individual. Many retrospective and prospective studies have studied factors predicting remission and relapse for children with GD. However, no stable predicting factors have been determined. Glaser et al. (14) showed that a high BMI score and smaller goiter size at the onset of diagnosis were predictors for remission, while Mussa et al. (26) showed that low TRab levels at the time of diagnosis and throughout the ATD treatment were related to remission. Similarly, Graser et al. (27) reported that a low sT3 level at the time of diagnosis and normalization of the thyroid functions within the first 3 months were affirmative predictors for remission. Kaguelidou et al*.* (28) found that relapse predictors were a younger age at diagnosis, severity of the disease at the onset of diagnosis, and ethnicity (being non-Caucasian). Ohye et al. (25) could not determine a statistically significant risk factor for remission or relapse. There were differences in sample size, analysis methods, and parameters measured among these studies. Those differences may have been responsible for the variety of the factors predicting remission and relapse. In this study, the initial ATD treatment duration and total ATD treatment duration parameters were statistically proved to be predictors of long-term remission. Other parameters were not found to be valuable as predictors of remission or relapse. Previous studies have shown that the clinical course is milder in patients who have TRab negativity at onset of the disease and small goiter size (29). In our study, the rate at which TRab became negative at the end of ATD treatment was similar between the remission and relapse groups (P = 0.602).

There is no consensus on the optimal ATD treatment duration to provide long-term remission of GD in children and adolescents. There is no sufficient evidence with regard to increasing the chance of remission in GD when using ATDs for more than 1 or 2 years in children and adolescents (30). It has been found that the remission rate after about 2 years of ATD treatment is lower than 30%, together with a high relapse rate (2,7,13). However, Léger et al.**(7) noted that a total 2-year ATD treatment in children was associated with a cumulative increase in the remission rate. Ohye et al. (25) reported a correlation between cumulative remission rate and the duration of treatment in 334 of 639 children and adolescents with GD. In our study initial ATD treatment duration and total ATD treatment duration were determined to be statistically longer in the remission group in comparison with the relapse group (P = 0.01, P = 0.03, respectively). Immune dysregulation induced by hyperthyroidism can be reinstated with euthyroidism. Therefore, chance of permanent remission of patients may be increased by staying in the euthyroid state for a long period (31). It is important when treating patients for a long duration to use the lowest possible ATD dose so as to increase the chance of remission and minimize the risk of side effects (32–34). 

There are studies suggesting that remission in GD with ATD treatment is independent of the type of drug (MMI or PTU) (28). Remission is independent of the additional use of levothyroxine. Propranolol, which is administered to alleviate the symptoms of hyperthyroidism, does not decrease the secretion of thyroid hormone and has no immunosuppressive effect. However, Codaccioni et al. (35) found long-term remission in 8 out of 26 (30.7%) patients treated only with propranolol. This rate is close to the spontaneous remission ratio expected (31%) in GD (36). This condition is explained by researchers as a spontaneous remission seen in the natural course of GD, as in other autoimmune diseases, and propranolol does not have a direct impact on remission. There were no patients treated with only propranolol in our study. Propranolol was administered in addition to ATD treatment in 35 (78%) patients. We did not detect a significant difference in terms of achieving remission in patients receiving propranolol. 

TSH receptor antibody positivity is important for diagnosis and follow-up of GD. It is commonly used as an assisted laboratory test in diagnosis of GD. A decrease in TRab level is expected due to reduced activity of the disease when in remission, and it is expected to increase in relapse based on increased disease activity (37). Contrary to what would be expected, TRab may not be positive at the onset of diagnosis, and TRab may become negative at the end of ATD treatment (35,38). In our study, TRab was negative for 9 patients at the onset of diagnosis, and 2 positive TRab values switched to negative at the end of ATD treatment in the relapse group. This discrepancy may be explained by the opinion that the TRab levels in circulation may not reflect the intrathyroidal TRab level, as discussed previously in the literature (35,38).

The medical treatment of GD may be carried out by dose-titration treatment or blockage-replacement treatment. It can be expected that a higher ATD dose will result in a higher rate of side effects. Since greater doses of ATD are required in blockage-replacement, evidence acquired from adults supports the opinion that dose-titration is the superior method (39). It is also a matter of debate whether blockage-replacement provides more remission in comparison with ATD treatment alone. It has been indicated in many studies that remission is independent of the dose of levothyroxine included in blockage-replacement (31,40). It has also been suggested that blockage-replacement decreases the frequency of follow-up required and provides longer periods of euthyroidism (8,38).

While 9 patients in our study received dose-titration treatment, 36 patients received blockage-replacement treatment. Remission and relapse rates were similar between these groups.

It has been reported that the rate of side effects due to ATD usage in GD is lower in children (5.1%–3%) than in adults (13.9%–51.9%) (41). In our study, the observed side effects were a skin rash (1 case) and an increase in liver function (1 case) (4.4%). Both patients were receiving PTU at the time. Even though no side effects from MMI treatment were observed in this study, patients treated with MMI should be carefully monitored for side effects such as liver dysfunction, skin reactions, and neutropenia. 

Radical treatment methods (RAI or surgery) should be taken into consideration in children when medical treatment has failed. Deciding to switch to definitive treatment requires not only taking into account the various prognostic factors that have been identified, but also considering the balance between individual risks and benefits based on the patient’s background and clinical course. It has been emphasized in a metaanalysis of adults that the optimal ATD treatment duration for GD is 12–18 months. If no remission is observed after this period, radical treatment options should be considered (39). The American Thyroid Association suggests radical treatment options for children in cases where remission cannot be attained following 2 years of ATD treatment (42). However, higher rates of remission are more probable with long periods of ATD treatment in children than adults (43), so radical treatment options should not be considered right away, even if recovery cannot be attained after 2 years of ATD treatment. We also determined that the length of total ATD treatment duration (42.14 ± 14.35 months) was a significant determining factor for remission. In our study, the average length of ATD treatment prior to usage of radical treatment was 3.5 years.

The most important problem that is observed in patients who receive radical treatment is the failure to adapt to long-term Na-L-thyroxine treatment. In addition, thyroidectomy has a higher complication rate in children than in adults. Haddow et al. revealed that 10 (30%) out of 33 patients treated with RAI experienced adaptation problems in long-term replacement treatment. These problems are an especially serious issue for women in the fertile period of their lives (44). In our study, 3 (2 surgery, 1 RAI) out of 6 patients whose received radical treatment continue to receive replacement treatment with Na-L-thyroxine. Therefore, remission should be attempted to be achieved with ATD prior to trying radical treatment. Families should be informed that ATD treatment may be long-lasting and that treatment with Na-L-thyroxine may be required for the remainder of the patient’s life in cases in which RAI or surgical treatment are received (40). 

In conclusion, ATD treatment is effective and reliable at achieving long-term remission of GD in children and adolescents. Because of this, children and adolescents with GD should be treated with long-term ATD before radical treatment. Radical treatment (RAI and surgery) should be considered as the last option and only in selected cases. In children and adolescents, the chance of long-term remission increases in proportion with the initial ATD treatment duration and the total ATD treatment duration. There is a need for further prospective randomized studies to evaluate the optimal treatment duration.
